# The asbestos–asbestosis exposure–response relationship: a cohort study of the general working population

**DOI:** 10.5271/sjweh.4153

**Published:** 2024-07-01

**Authors:** Inge Brosbøl Iversen, Jesper Medom Vestergaard, Johan Ohlander, Susan Peters, Elisabeth Bendstrup, Jens Peter E Bonde, Vivi Schlünssen, Jakob H Bønløkke, Finn Rasmussen, Zara A Stokholm, Michael B Andersen, Hans Kromhout, Henrik A Kolstad

**Affiliations:** 1 Department of Occupational Medicine, Danish Ramazzini Centre, Aarhus University Hospital, Aarhus, Denmark.; 2 Institute for Risk Assessment Sciences, Utrecht University, Utrecht, The Netherlands.; 3 Center for Rare Lung Diseases, Department of Respiratory Diseases and Allergy, Aarhus University Hospital, Aarhus, Denmark.; 4 Department of Occupational and Environmental Medicine, Bispebjerg and Frederiksberg Hospital, Copenhagen, Denmark.; 5 Department of Public Health, Danish Ramazzini Centre, Aarhus University, Aarhus, Denmark.; 6 Department of Occupational and Environmental Medicine, Danish Ramazzini Centre, Aalborg University Hospital, Aalborg, Denmark.; 7 Department of Radiology, Aarhus University Hospital, Aarhus, Denmark.; 8 Department of Radiology, Copenhagen University Hospital Herlev and Gentofte, Copenhagen, Denmark.; 9 Department of Clinical Medicine, Copenhagen University, Copenhagen, Denmark.; 10 Department of Clinical Medicine, Aarhus University, Aarhus, Denmark.

**Keywords:** interstitial lung disease, job exposure matrix, occupational exposure, register study

## Abstract

**Objectives:**

The association between asbestos exposure and asbestosis in high-exposed industrial cohorts is well-known, but there is a lack of knowledge about the exposure–response relationship for asbestosis in a general working population setting. We examined the exposure–response relationship between occupational asbestos exposure and asbestosis in asbestos-exposed workers of the Danish general working population.

**Methods:**

We followed all asbestos-exposed workers from 1979 to 2015 and identified incident cases of asbestosis using the Danish National Patient Register. Individual asbestos exposure was estimated with a quantitative job exposure matrix (SYN-JEM) from 1976 onwards and back-extrapolated to age 16 for those exposed in 1976. Exposure–response relations for cumulative exposure and other exposure metrics were analyzed using a discrete time hazard model and adjusted for potential confounders.

**Results:**

The range of cumulative exposure in the population was 0.001 to 18 fibers per milliliter-year (f/ml-year). We found increasing incidence rate ratios (IRR) of asbestosis with increasing cumulative asbestos exposure with a fully adjusted IRR per 1 f/ml-years of 1.18 [95% confidence interval (CI) 1.15– -1.22]. The IRR was 1.94 (95% CI 1.53–2.47) in the highest compared to the lowest exposure tertile. We similarly observed increasing risk with increasing cumulative exposure in the inception population.

**Conclusions:**

This study found exposure–response relations between cumulative asbestos exposure and incident asbestosis in the Danish general working population with mainly low-level exposed occupations, but there is some uncertainty regarding the exposure levels.

Asbestos has been used for thousands of years due to advantageous properties such as strength, heat resistance and durability (1). It is a group of naturally occurring silicate fibers with chrysotile being the fiber type primarily used in Denmark (2). Approximately 620 000 tons of asbestos have been consumed in Denmark over the years, mainly for production of asbestos-cement products, with consumption peaking in the 1970s (3). Since the 1980s, many countries have introduced bans on asbestos use due to its negative health impact (4). A national ban was imposed in Denmark in 1986 (4). Despite the bans, occupational exposure to asbestos still occurs during demolition, repair and maintenance work of asbestos-containing material especially in the construction industry (5, 6). Furthermore, a number of countries without bans still have significant asbestos production and consumption today with an estimated 125 million people globally exposed to asbestos at work (7).

When inhaled, asbestos fibers are deposited in the lungs where they stimulate macrophages and other immune cells to produce mediators that stimulate fibroblast proliferation and collagen synthesis, which can ultimately result in fibrosis (8).

The recognition of an association between asbestos exposure and pulmonary fibrosis is often dated back to around 1930, but it had already been suggested prior to this (9). Cases of pulmonary fibrosis related to asbestos exposure are named asbestosis, and incidence of this disease is still increasing at the global level (10). Diagnosis is based on typical clinical, radiological or histological findings and a history of asbestos exposure (11, 12). Pulmonary fibrosis due to asbestosis may be indistinguishable from other interstitial lung diseases such as idiopathic pulmonary fibrosis (IPF) (13). Exposure–response relationships between cumulative asbestos exposure and asbestosis have been observed in high-exposed asbestos industry cohorts (14–17). Studies of asbestosis mortality and incidence in the general working population have observed increased rates of asbestosis in a number of other occupations, such as in construction, the metal industry and for workers in electric power, gas and water utilities (18, 19). But there is a lack of knowledge about exposure–response relationships for asbestosis in a general working population setting, as these studies do not include a quantitative exposure assessment.

The aim of the present study was to examine the exposure–response relationship of incident asbestosis following occupational asbestos exposure in a large general working population including all asbestos-exposed workers in Denmark 1976–2015.

## Methods

The study was registered at the repository of the Central Denmark Region (j.no.: 1-16-02-196-17). Studies in Denmark without biological materials do not need approval from the Committee of Health Research Ethics.

For an overview of the setting and data sources of the study, see the supplementary material (www.sjweh.fi/article/4153, appendix 1).

### Study population

The study population is derived from the DOC*X cohort of all gainfully employed workers in Denmark since 1976, which contains annual information on occupation coded according to the 1988 International Standard Classification of Occupations (ISCO-88) (20). When an ISCO-88 code was missing for a given year, we assigned the latest valid code up to 5 years back.

In the study population, we included all workers born 1900 or later with at least one year of employment in an occupation with asbestos exposure according to the job exposure matrix (JEM) SYN-JEM (described in the Methods section). Within the main study population, we defined an inception cohort of workers born 1956 or later with complete work histories since age 20. Complete work history since age 16 would have been preferable, but such a population would yield too few cases for meaningful analyses.

### Asbestosis

We identified cases by first recorded diagnosis of asbestosis [International Classification of Diseases (ICD) version 8 code 515.2 and ICD version 10 code J61] in the Danish National Patient Register (21), which contains information on all inpatient contacts in Denmark since 1977 and outpatient contacts since 2015.

IPF may exhibit radiological and pathological characteristics similar to asbestosis. We therefore performed a supplementary analysis for this outcome to assess possible diagnostic bias. IPF could not be identified in ICD-8 and was therefore defined by ICD-10 code J84.1A and only from 1994–2015, as ICD-10 was introduced in Denmark in 1994.

### Asbestos exposure assessment

The original version of SYN-JEM (22) contained annual full-shift geometric mean exposure intensity estimates for asbestos for all ISCO-68 job codes, which has more detail than ISCO-88, and was based on 27 958 personal exposure measurements from Europe and Canada. In order to create the ISCO-88 version of SYN-JEM, the exposure database was recoded to ISCO-88 codes and remodeled. German measurements from the MEGA database were not available, so the ISCO-88 version of SYN-JEM is based on 21 946 personal exposure measurements. SYN-JEM assigns an annual trend of -10.8% for all years from 1975 onward. We used the exposure estimates for northern European countries to estimate exposure.

For each registered year of employment in 1976–2015, we linked information on occupation from the DOC*X cohort with an ISCO-88 version of the SYN-JEM (22) to estimate annual intensity of asbestos exposure. For all workers exposed to asbestos in 1976, we extrapolated exposure back to age 16. We assumed no trend before 1975 to avoid extrapolation to years without available measurements resulting in unrealistically high exposure estimates. We created the following exposure metrics for each worker: (i) cumulative exposure (fibers per milliliter (f/ml)-year) as the sum of the annual exposure intensities; (ii) highest attained exposure intensity (f/ml); and (iii) duration of exposure (years).

### Statistical analyses

We started follow-up the year following the first year of employment in an asbestos-exposed occupation, as month or day of employment was not recorded. First possible year of follow-up was 1979, two years after the first year of registrations in the National Patient Register as we applied a 2-year washout period 1977–78 to reduce the number of prevalent cases. We followed workers until first year of an asbestosis diagnosis, death, emigration, disappearance or end of follow-up on 31 December 2015, whichever came first. Incidence rates of asbestosis across the follow-up period were explored. We estimated the percentage of person-years in the total study population with missing information on occupation due to work history data only being available from 1976 (see supplementary appendix 2).

We used a discrete time hazard model with person-years as unit of analysis, yielding incidence rate ratios (IRR) with 95% confidence intervals (CI) (23). Exposure metrics were categorized into tertiles based on the person-year distributions. We applied two models for adjustment: model 1 was adjusted for age, sex and calendar year of follow-up, while model 2 was additionally adjusted for education, lifestyle JEM estimates of smoking, previously diagnosed connective tissue disease, fibrogenic medications and cumulative silica and organic dust exposure. Supplementary appendix 3 provides rationale and categorizations of covariates. All variables were treated as time-varying.

In the supplementary analyses, we stratified for sex, calendar year of follow-up and birth year. We had to reduce the number of covariates included in some of the stratified analyses to make the models fit. The included covariates are reported in the tables.

We fitted restricted cubic splines with 95% CI for cumulative asbestos exposure as a continuous variable and placed knots at the 5, 50 and 95 percentiles (24). Plots were truncated at the 99 percentile.

Exposure tertiles were recalculated for the inception population.

We used Stata 17 (StataCorp, College Station, TX, USA) for all analyses.

## Results

The total study population consisted of 1 514 136 workers ever employed in occupations with asbestos exposure who accumulated 33 878 817 person-years, and we identified 1084 incident cases of asbestosis from 1979 onwards. Details of the establishment of the study population can be found in supplementary figure S1. The inception population comprised 901 957 workers with a total of 18 024 939 person-years and 17 cases of asbestosis.

Cumulative exposure ranged from 0.001–18 f/ml-year. Increasing cumulative asbestos exposure was associated with male sex, being skilled blue-collar worker, lower level of education, higher probability of smoking and high levels of cumulative organic dust exposure (table 1). Being unemployed or retired was also associated with increasing cumulative exposure, which is expected as retired workers are likely to be older and thus have been exposed during earlier years when exposure intensity was higher.

**Table 1:** Distribution of person-years (PY) at risk (%) among 1 514 136 workers, Denmark, 1979–2015.

		Cumulative asbestos exposure (f/ml-years)				
		0.001–0.026 (N=11 295 055)		0.027–0.14 (N=11 259 428)		0.15–18 (N=11 324 334)
		PY		PY		PY
Sex						
	Male	70.1		68.3		77.7
	Female	29.9		31.7		22.3
Occupation ^a^						
	Armed forces	3.7		2.2		2.3
	White-collar	32.7		24.2		15.3
	Skilled blue-collar	18.4		19.7		28.3
	Unskilled blue-collar	26.6		31.3		24.0
	Unemployed or retired	17.1		20.6		28.7
	Missing	1.5		2.0		1.4
Age (years)						
	<20	4.2		1.2		0.0
	20–24	16.5		5.9		1.4
	25–29	16.8		9.9		4.2
	30–34	14.8		12.4		6.7
	35–39	12.7		13.6		8.9
	40–44	10.1		13.4		10.9
	45–49	7.1		11.9		12.6
	50–54	5.2		9.2		13.1
	55–59	4.3		7.1		12.2
	60–64	3.4		5.7		10.6
	65–69	2.4		4.2		8.2
	70–74	1.4		2.7		5.5
	75–79	0.7		1.6		3.3
	≥80	0.4		1.2		2.4
Calendar year						
	1979–84	0.6		9.6		9.4
	1985–94	14.0		24.3		31.6
	1995–2004	36.6		27.0		30.3
	2005–15	48.8		39.1		28.7
Probability of smoking (%)						
	5–30	46.5		33.3		20.2
	31–45	39.9		28.9		31.1
	46–74	13.6		37.7		48.7
Education						
	Unknown	2.0		3.3		4.6
	Lower secondary	34.2		43.1		37.7
	Vocational / high secondary	46.2		42.6		48.1
	Short cycle higher	4.3		3.3		5.1
	Medium cycle higher	9.3		5.8		3.6
	Long cycle higher	4.1		1.9		0.9
Previous connective tissue disease ^b^						
	No	99.3		99.1		98.9
	Yes	0.7		0.9		1.1
Fibrogenic medication ^b^						
	No	97.6		97.7		97.7
	Yes	2.4		2.3		2.3
Cumulative organic dust exposure						
	0	38.6		35.2		40.0
	1^st^ tertile	22.7		16.1		7.1
	2^nd^ tertile	25.6		25.5		23.4
	3^rd^ tertile	13.2		23.2		29.5
Cumulative respirable crystalline silica exposure						
	0	83.3		79.5		87.0
	1^st^ tertile	9.3		5.2		2.2
	2^nd^ tertile	4.9		8.4		3.4
	3^rd^ tertile	2.4		6.9		7.3
Duration of exposure (years)						
	1–2	57.1		30.2		0.0
	3–8	41.2		40.8		26.6
	9–70	1.7		29.0		73.3

^a^ Grouped according to International Standard Classification of Occupations (ISCO), 1988 revision: Armed forces (ISCO-88 codes 0110), white-collar workers (ISCO-88 codes 1000-5999), skilled blue-collar workers (ISCO-88 codes 6000-7999), unskilled blue-collar workers (ISCO-88 codes 8000-9999), others (unemployed or retired). ^b^ As defined in the covariates section in the online supplementary material.

We observed increasing risk of asbestosis with increasing cumulative asbestos exposure in the partially and the fully adjusted models; the latter showing an IRR of 1.18 per 1 f/ml-years (95% CI 1.15–1.22) and an IRR of 1.94 (95% CI 1.53–2.47) for the highest compared with the lowest exposed tertile (table 2). For highest attained exposure, we also observed increasing risk with increasing exposure [fully adjusted IRR 1.09 (95% CI 1.08–1.11) per 0.01 f/ml]. The IRR for the highest compared with the lowest exposed tertile was 2.35 (95% CI 1.78–3.11). For duration of exposure, the fully adjusted IRR was 1.06 per five years (95% CI 1.04–1.08) and 1.43 (95% CI 1.18–1.75) for the highest compared with the lowest exposed tertile.

**Table 2:** Incidence rates (IR) and incidence rate ratios (IRR) of asbestosis following exposure to asbestos, N=1 514 136 workers, 1979–2015, Denmark. [PY=person years; CI=confidence interval.]

Exposure		Asbestosis				
		PY	Cases	IR ^a^	IRR (95% CI) ^b^	IRR (95% CI) ^c^
Cumulative exposure (f/ml-years)						
	0.001–0.026	11 295 055	83	0.73	1	1
	0.027–0.14	11 259 428	200	1.78	1.14 (0.88–1.48)	1.11 (0.85–1.44)
	0.15–18	11 324 334	801	7.1	2.08 (1.65–2.63)	1.94 (1.53–2.47)
	Per 1 f/ml–years				1.14 (1.11–1.17)	1.18 (1.15–1.22)
Highest attained exposure (f/ml)						
	0.001–0.005	11 287 927	61	0.54	1	1
	0.006–0.047	11 176 134	410	3.67	1.80 (1.37–2.37)	1.68 (1.27–2.23)
	0.048–0.34	11 414 756	613	5.37	2.44 (1.86–3.19)	2.35 (1.78–3.11)
	Per 0.01 f/ml				1.08 (1.07–1.10)	1.09 (1.08–1.11)
Duration (years)						
	1–2	9 856 286	137	1.39	1	1
	3–8	12 266 425	253	2.06	1.09 (0.88–1.34)	0.99 (0.79–1.23)
	9–70	11 756 106	694	5.9	1.46 (1.21–1.75)	1.43 (1.18–1.75)
	Per 5 years				1.05 (1.03–1.07)	1.06 (1.04–1.08)

^a^ Unadjusted, per 100 000 PY. ^b^ Adjusted for age, sex and calendar year. ^c^ Adjusted for age, sex, calendar year, education, probability of smoking, connective tissue disease, medications, cumulative exposure to respirable crystalline silica and cumulative exposure to organic dust.

**Figure 1:**
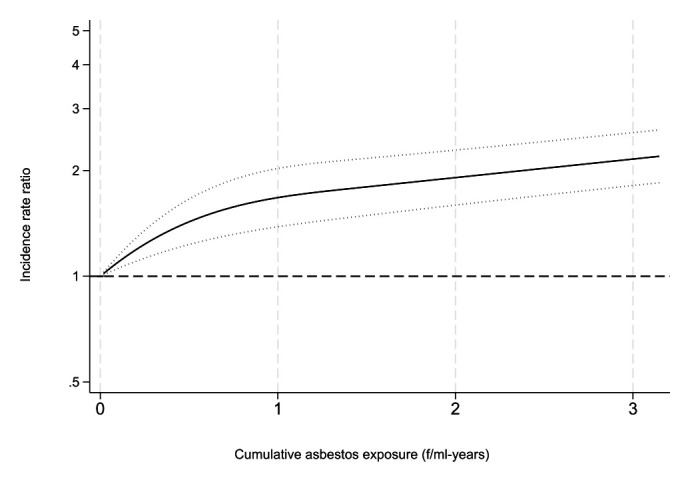
Restricted cubic spline fits of adjusted incidence rate ratios of asbestosis by cumulative asbestos exposure. Dotted lines mark 95% confidence interval.

Restricted cubic spline analysis showed increasing risk of asbestosis with increasing cumulative asbestos exposure. The increase was strongest up to around 1 f/ml-years (figure 1).

In analyses stratified by sex, we observed increasing risk of asbestosis with increasing cumulative exposure for men but not women (supplementary table S1). Only 20 cases of asbestosis were diagnosed among women. Increasing IRR with increasing cumulative asbestos exposure was observed without marked differences in risk estimates for workers born both 1900–1940 and after 1940 (supplementary table S2). Stratification by calendar year of follow-up yielded similar results for 1979–1994 and 1995–2015 (supplementary table S3).

Estimates for the inception population were based on only 17 cases of asbestosis, corresponding to an unadjusted incidence rate of 0.09 per 100 000 person-years. Similar to the total study population, we found increasing risk of asbestosis with increasing cumulative exposure with a fully adjusted IRR of 1.17 (95% CI 0.94–1.45) per 0.1 f/ml-years. IRR was 1.25 (0.31–5.07) for the highest compared with the lowest exposure tertile (supplementary table S4).

For IPF, we did not observe any trend for any of the exposure metrics. For cumulative exposure, we observed a fully adjusted IRR of 0.97 (0.76–1.24) per 1 f/ml-years (supplementary table S5).

Construction, the metal industry and motor vehicle and other mechanics accounted for >50% of all exposed person-years (supplementary table S6). Incidence rates of asbestosis showed no obvious trend but peaked in 1990–1994 (supplementary table S7). Information on occupation was missing for 23% of all potentially employed years from age 16 in the total study population due to no information on occupation being available prior to 1976.

## Discussion

In this large general working population, we observed increasing risk of incident asbestosis with increasing cumulative asbestos exposure. Findings were similar in the inception population with complete work history, but they were based on few cases and had wide CI.

It is well-known that an exposure–response relation between asbestos exposure and risk of asbestosis exists at high levels of asbestos exposure in asbestos mining and processing factories (14–17, 25). However, our study population is not comparable to those in studies of high-exposed occupations as asbestos mining has been absent and processing limited in the Danish general working population. Instead, most person-years with asbestos exposure have occurred in construction, the metal industry and among motor vehicle and other mechanics.

Previous general population studies of asbestosis have investigated incidence or mortality of asbestosis, but without quantitative exposure data. DeBono et al (19) observed increased incidence rates of asbestosis in construction, in the metal industry, and for workers in electric power, gas and water utilities, while other studies found increased mortality rates of asbestosis in a number of occupations in the construction industry, and among metal workers, electrical plant operators and chemical workers (18, 26). In occupation- or industry-specific studies, Imbernon et al (27) observed exposure–response relationships between cumulative asbestos exposure and asbestosis in the electricity and gas industry. Other studies without quantification of exposure have observed increased risk of incident asbestosis among motor vehicle mechanics (28) and increased mortality rates among carpenters, insulators and plumbers and pipefitters (29–31). Thus, our findings confirm that occupations outside the highest-exposed industries are also at risk of asbestosis by demonstrating exposure–response relationships in a large general working population including all asbestos-exposed occupations in Denmark.

### Strengths and limitations

We identified cases of asbestosis using health registers that cover all hospital contacts in Denmark. The public Danish healthcare services are free of charge but we cannot rule out that high asbestos exposure may have increased the likelihood of referral to a hospital. We introduced a wash-out period to reduce the number of prevalent cases. Additionally, we performed analyses stratified by calendar year, which showed similar risk patterns in the early and later years of the follow-up period.

We have no information on the specific diagnostic process leading to asbestosis diagnosis for the individual cases, but in Denmark comprehensive diagnostic programs are carried out at four national interstitial lung disease centers for all suspected cases of interstitial lung disease. The program encompasses a detailed medical history, clinical examination including pulmonary function tests with diffusion capacity for carbon monoxide tests and a high-resolution CT scan. All patients are discussed at a multidisciplinary team conference, and if required, a lung biopsy is performed with subsequent reevaluation of the case at a multidisciplinary team conference. Guidelines from the Danish Respiratory Society set up three main criteria for the diagnosis: sufficient asbestos exposure (with no specification), characteristic radiological findings, and exclusion of other similar diseases (32). Interpretation of these criteria may have differed during follow-up, but we did not observe a tendency of increasing incidence of asbestosis which could have signaled a relaxation of the approach to the criteria over the years.

Radiological and pathological findings for asbestosis may appear similar to other interstitial lung diseases, most importantly IPF (13). If other interstitial lung diseases that are not associated with asbestos exposure are misclassified as asbestosis – and this happens more often in high- than low-exposed cases, which could be motivated by worker’s compensation being available for asbestosis but not IPF – it may lead to inflation of the risk estimates. On the other hand, we observed no decline in risk of IPF with increasing asbestos exposure, which does not support exposure-dependent misclassification. The problem of diagnostic misclassification arises because these diseases are partly defined by the presence or absence of an exposure and not only by disease characteristics. This in turn obstructs causal analyses as the association is pre-defined.

Information on occupation was only available from 1976 onwards, meaning we lacked information for an estimated 23% of all potentially employed years in the total study population so that a considerable asbestos exposure for workers with employment prior to 1976 is unknown. Therefore, for workers with prior exposure, the actual cumulative exposure would have been higher than the exposure to which they were assigned, leading to a likely substantial underestimation of cumulative exposure. We addressed the issue of missing work history by back-extrapolating exposure to age 16 for all workers exposed in 1976 to achieve more realistic cumulative exposure levels. We assumed no trend in exposure for years before 1975, leading to conservative exposure estimates that are likely to result in an underestimation of cumulative exposure levels. On the other hand, duration of exposure will likely be overestimated, thereby potentially leading to overestimation of cumulative exposure levels. The inception population is largely unaffected by the missing work history, even though complete work history from age 16 would have been preferable, and the fact that analyses of the inception population produced risk estimates comparable to those in the primary analyses, support our findings in the total study population. Analyses of the inception population also addresses the issue of workers already employed at the start of follow-up, which may as a whole be a survivor population less susceptible to the effects of asbestos exposure than all who worked before the beginning of follow-up. In the main study population, we observed an overrepresentation of the unemployed or retired group in the highest exposure tertile, which could be a sign of a healthy worker survivor effect, where the highest-exposed workers are more likely to leave the job or retire. This would, however, lead to underestimation of the exposure–response relationship.

A major strength of this study is that it was the first with quantitative exposure data for a general working population. We used a quantitative JEM that has previously confirmed known associations for lung cancer and mesothelioma (33, 34) to estimate individual asbestos exposure levels in the Danish general working population where most asbestos-exposed workers have been employed in lower-exposed occupations such as in construction. By using the ISCO-88 version of SYN-JEM, assigned exposure intensities will have been lower than would have been the case when using the ISCO-68 version of SYN-JEM, due to less-specific and fewer job categories in ISCO-88 compared to ISCO-68, leading to lower cumulative exposure levels. SYN-JEM also estimates geometric and not arithmetic means of exposure. The use of arithmetic means would have resulted in approximately 60% higher cumulative exposure levels. Using JEM-based exposure estimates entails a risk of non-differential misclassification as the matrix does not allow for variation in exposure within an occupation, but this will primarily lead to Berkson-type error, which results in increased uncertainty of risk estimates but does not attenuate exposure–response relationships (35). However, the use of a JEM when investigating a disease defined by exposure, such as asbestosis, may constitute a problem as the most-exposed workers in any given occupation are also the most likely to be diagnosed with asbestosis. This results in JEM-produced mean estimates not being representative for diagnosed cases, which may lead to inflation of the exposure–response relationship. This problem, as well as the issue of diagnostic misclassification, can be avoided by studying the disease phenotype independent of exposure, for instance by using lung scan analysis software.

Even though asbestos should, by definition, be the only cause of asbestosis, we adjusted for use of fibrogenic medications, prior connective tissue disease, smoking and other occupational dust exposures that are known causes of pulmonary fibrosis (36). Adjustment for smoking was especially relevant, as we observed a very skewed distribution of probability of smoking by cumulative exposure level. In most cases, supplementing adjustment for age, sex and calendar year with these factors attenuated the risk estimates. We used a lifestyle JEM that has previously performed well (37) to adjust for smoking. Omitting adjustment for smoking did not lead to significant changes to our results.

### Concluding remarks

This study found exposure–response relationships between cumulative asbestos exposure and incident asbestosis in the general Danish working population, providing evidence of increased risk of asbestosis outside high-exposed industries. Work histories were missing prior to 1976, which we addressed by back-extrapolation of exposure, but some uncertainty regarding the cumulative exposure estimates remains. Defining asbestosis by asbestos exposure is a major challenge when interpreting these findings that can be circumvented by studying lung imaging and other clinical findings blinded to exposure status.

## Supplementary material

Supplementary material
