# Supplementary material for: The asbestos–asbestosis exposure–response relationship: a cohort study of the general working population
Source: Scand J Work Environ Health. 2024 Jun 27;50(5):372–9. doi: 10.5271/sjweh.4153 (PMC11245331; doi:10.5271/sjweh.4153)
Supplement: Supplementary material [file SJWEH-50-372-S001.pdf]

The asbestos–asbestosis exposure–response relationship: a cohort study of the general working population[1]

by Inge Brosbøl Iversen, PhD,[2] Jesper Medom Vestergaard, MIT, Johan Ohlander, PhD, Susan Peters, PhD, Elisabeth Bendstrup, PhD, Jens Peter E Bonde, DMSc, Vivi Schlünssen, PhD, Jakob H Bønløkke, PhD, Finn Rasmussen, DMSc, Zara A Stokholm, PhD, Michael B Andersen, PhD, Hans Kromhout, PhD, Henrik A Kolstad, PhD

1. *Supplementary material*
2. *Correspondence to: Dr Inge Brosbøl Iversen, Department of Occupational Medicine, Danish Ramazzini Centre, Aarhus University Hospital, Palle Juul-Jensens Boulevard 99, 8200 Aarhus N, Denmark. [E-mail: iniver@rm.dk].*

Contents

Appendix 1:	Setting and data sources
Appendix 2:	Estimation of percentage of years with missing information on occupation
Appendix 3:	Description of covariates
Table S1:	Analyses stratified by sex
Table S2:	Analyses stratified by birth year
Table S3:	Analyses stratified by calendar year of follow-up
Table S4:	Analyses in inception population
Table S5:	Analyses of idiopathic pulmonary fibrosis
Table S6:	Distribution of person-years at risk by ISCO-88 codes
Table S7:	Incidence rates of asbestosis during follow-up
Figure S1:	Flowchart of the establishment of the study population

Appendix 1

Setting and data sources

Denmark has a universal tax-funded healthcare system. All Danish residents are assigned a personal identification number, allowing linkage of national registries at an individual level and complete follow-up.

Our study made use of the following data sources:

- Danish National Patient Register (1), which contains information on all inpatient (from 1977) and outpatient (from 1995) contacts with Danish public hospitals. Diagnoses are coded according to the 8th (1977-1993) and 10th (1994-2015) versions of the International Classification of Diseases (ICD-8 and ICD-10).
- Danish National Prescription Registry (2), which contains data on all dispensed prescriptions from community pharmacies since 1994.
- Statistics Denmark's education registry, which contains information on highest attained education.
- Danish Civil Registration System, which records information on birth date, sex and vital status since 1968.
- Danish Occupational Cohort (DOC*X), which contains which contains annual information on occupation coded according to the 1988 International Standard Classification of Occupations (ISCO-88) (3), for all Danish residents with at least one month of paid employment from 1976 to 2015 (4).

Appendix 2

Information on occupation for years of employment prior to 1976 is not registered in the DOC*X.

We assumed an age of 16 years at first employment and a retirement age of 67 if end of follow-up (EOF) due to another reason was not registered earlier.

We calculated the difference in years between age 16 and 1976 for all workers born before 1906 and summed these differences to provide the number of potentially exposed person-years with missing information on occupation. Maximum number of years with missing information on occupation is 60 years for persons born in 1900.

Number of potentially exposed person-years where information on occupation was available included all years 1976-2015 for those born 1960 or later. For workers born before 1960, we calculated the difference between 1976 and EOF or retirement and summed these differences.

The percentage of years with missing information on occupation was calculated by dividing years with missing information by the total number of potentially exposed person-years in the study population.

Appendix 3

Definition of covariates		
**Covariate**	**Categories**	**Data source**
Age	<50, 50-54, 55-59, 60-64, 65-69, 70-74, 75-79, ≥80 years<br>In inception population: <35, 35-39, 40-44, 45-49, ≥50 years	Population register
Sex	Male, female	Population register
Calendar year of follow-up	1977-84, 1985-94, 1995-2004, 2005-15	
Educational level	Lower secondary, vocational or higher secondary, short, medium, and long cycle higher education and unknown	Highest attained education according to Statistics Denmark's education registry
Connective tissue disease	Yes, no	National Patient Register by ICD-8 codes 446, 712, 716 and 734 and ICD-10 codes M05, M06, M08, M09 and M30-M36
Fibrogenic medications	Yes, no	National Prescription Registry by ATC codes Antineoplastic and immunomodulating agents (all ATC codes with first letter L), nitrofuran derivatives (ATC code J01XE), and amiodarone (C01BD01).
Cumulative silica exposure	0, 1st tertile, 2nd tertile, 3rd tertile	Estimated using SYN-JEM ISCO-88 estimates for respirable crystalline silica.
Cumulative organic dust exposure	0, 1st tertile, 2nd tertile, 3rd tertile	Estimated using ALOHA+JEM estimates for organic dust exposure.
Probability of smoking	Tertiles: 5-30, 31-45, 46-74%	Estimated using a smoking job exposure matrix developed for the DOC*X cohort

Silica and organic dust: SYN-JEM provides quantitative estimates of annual silica exposure (5) while the ALOHA+ JEM assigns semi-quantitative exposure ratings (0=none, 1=low, 2=high) and the annual exposure intensities is calculated as the squared ALOHA+ JEM estimates, corresponding to 0, 1 and 4 (6). Annual exposure was summed to yield cumulative silica and organic dust exposure, respectively, which was then divided into tertiles for the analyses.

Smoking probability: The smoking job exposure matrix is based on Danish survey information on smoking (7). The JEM contains sex- and calendar year-specific estimates of smoking prevalence for

all ISCO-88 occupations and predicts mortality and acute myocardial infarction independent of other determinants as expected (4, 8). We assigned years without employment the same smoking probability as in the latest previous year with employment. If there was no previous information on smoking probability available due to ISCO-88 codes that could not be linked with the smoking JEM, we assigned the same smoking habit as in the next later period where smoking habit could be assigned.

All covariates were decided upon a priori based on a review of the literature and the availability of relevant information in the registers (9-13).

Table S1. Incidence rate ratios (IRR) of asbestosis following occupational exposure to asbestos, stratified by sex, 1,514,136 workers, 1979-2015

	Men			Women		
Exposure	Person-years	Cases	IRR (95% CI)[a]	Person-years	Cases	IRR (95% CI)
Cumulative exposure (f/ml-years)						
0.001-0.026	7,915,220	81	1	3,379,835	n.r.[b]	1
0.027-0.14	7,688,860	191	1.24 (0.95-1.62)	3,570,568	n.r.[b]	1.88 (0.39-9.13)
0.15-18	8,797,336	792	2.42 (1.91-3.07)	2,526,998	n.r.[b]	1.45 (0.30-7.04)
Per 1 f/ml-years			1.15 (1.12-1.18)			0.76 (0.10-6.00)

[a]Adjusted for age, sex and calendar year

[b]Total number of asbestosis cases among women: 20

Table S2. Incidence rate ratios (IRR) of asbestosis following exposure to asbestos, stratified by birth year, 1,514,136 workers, 1979-2015

	Birth year 1900-1940				Birth year >1940			
Exposure	Person-years	Cases	IRR (95% CI)[a]	IRR (95% CI)[b]	Person-years	Cases	IRR (95% CI)[a]	IRR (95% CI)[b]
Cumulative exposure (f/ml-years)								
0.001-0.026	616,620	46	1	1	10,678,435	37	1	1
0.027-0.14	1,781,186	157	1.24 (0.89-1.73)	1.14 (0.81-1.59)	9,478,242	43	0.96 (0.62-1.50)	0.97 (0.62-1.53)
0.15-18	34,420,332	648	2.20 (1.63-2.97)	2.04 (1.49-2.79)	7,882,002	153	1.86 (1.29-2.70)	1.88 (1.28-2.76)
Per 1 f/ml-years			1.14 (1.11-1.18)	1.17 (1.13-1.21)			1.46 (1.28-1.67)	1.46 (1.28-1.67)

[a]Adjusted for age, sex and calendar year

[b]Adjusted for age, sex, calendar year, probability of smoking, connective tissue disease, medications, cumulative exposure to respirable crystalline silica and cumulative exposure to organic dust

Table S3. Incidence rate ratios (IRR) of asbestosis following exposure to asbestos, stratified by calendar year of follow-up, 1,514,136 workers, 1979-2015

	1979-1994			1995-2015		
Exposure	Person-years	Cases	IRR (95% CI)[a]	Person-years	Cases	IRR (95% CI)[a]
Cumulative exposure (f/ml-years)						
0.001-0.026	1,650,409	18	1	9,644,646	65	1
0.027-0.14	3,808,117	96	1.20 (0.72-1.98)	7,451,311	104	1.08 (0.79-1.48)
0.15-18	4,645,436	343	1.95 (1.21-3.14)	6,678,898	458	2.27 (1.74-2.96)
Per 1 f/ml-years			1.08 (1.03-1.12)			1.25 (1.20-1.30)

[a]Adjusted for age and sex

Table S4. Incidence rate ratios (IRR) of 17 cases of asbestosis following exposure to asbestos, 901,957 workers with complete work histories since age 20 (inception cohort), 1979-2015, Denmark

	Asbestosis	
Exposure	IRR (95% CI)*	IRR (95%CI) †
Cumulative exposure (f/ml-years)		
0.001-0.012	1	1
0.013-0.054	0.75 (0.15-3.77)	0.75 (0.15-3.78)
0.055-2.3	1.28 (0.32-5.17)	1.25 (0.31-5.07)
Per 0.1 f/ml-years	1.17 (0.95-1.44)	1.17 (0.94-1.45)

*Adjusted for age, sex and calendar year

†Adjusted for age, sex, calendar year and probability of smoking (number of covariates reduced to make models fit)

Table S5. Incidence rate ratios (IRR) of idiopathic pulmonary fibrosis (IPF) following asbestos exposure among 1,456,658 workers, Denmark, 1994-2015*

	IPF			
Exposure	Person-years	Cases	IRR (95% CI) †	IRR (95% CI) ‡
Cumulative exposure (f/ml-years)				
0.001-0.027	10,022,964	23	1	1
0.028-0.14	7,495,803	35	1.20 (0.70-2.04)	1.26 (0.72-2.20)
0.15-1.9	7,194,336	62	1.18 (0.71-1.93)	1.17 (0.69-1.99)
Per 0.1 f/ml-years			0.99 (0.93-1.05)	0.98 (0.92-1.04)

*In this analysis, follow-up is restricted to 1994-2015, as IPF can only be defined using ICD-10 which was used in Denmark from 1994.

†Adjusted for age, sex and calendar year

‡Adjusted for age, sex, calendar year, probability of smoking, connective tissue disease, fibrogenic medications, exposure to respirable crystalline silica and exposure to organic dust

Table S6. Distribution of exposed person-years at risk by ISCO-88 codes in the total study population.

ISCO-88	Description	Person-years
0110	Armed forces	917,848
2147	Mining engineers, metallurgists and related professionals	5,790
3117	Mining and metallurgical technicians	8,372
3140	Ship and aircraft controllers and technicians	18,992
3141	Ships' engineers	58,041
3142	Ships' deck officers and pilots	112,931
3150	Safety and quality inspectors	162
3151	Building and fire inspectors	38,367
3152	Safety, health and quality inspectors	86,908
5161	Fire-fighters	42,674
7100	Extraction and building trades workers	62,195
7110	Miners, shotfirers, stone cutters and carvers	215
7111	Miners and quarry workers	719
7112	Shotfirers and blasters	313
7121	Builders, traditional materials	12,493
7129	Building frame and related trades workers not elsewhere classified	60,186
7130	Building finishers and related trades workers	44,072
7131	Roofers	28,440
7132	Floor layers and tile setters	26,690
7133	Plasterers	5,461
7134	Insulation workers	31,053
7136	Plumbers and pipe fitters	399,349
7137	Building and related electricians	570,738
7140	Painters, building structure cleaners and related trades workers	587
7141	Painters and related workers	560,263
7143	Building structure cleaners	30,430
7200	Metal, machinery and related trade workers	732,915
7214	Structural-metal preparers and erectors	147,595
7215	Riggers and cable splicers	4,214
7220	Blacksmiths, tool-makers and related trade workers	2,324
7221	Blacksmiths, hammer-smiths and forging-press workers	284,223
7222	Tool-makers and related workers	252,335
7224	Metal wheel-grinders, polishers and tool sharpeners	17,055
7230	Machinery mechanics and fitters	9,799
7231	Motor vehicle mechanics and fitters	625,938
7232	Aircraft engine mechanics and fitters	28,646
7233	Agricultural- or industrial-machinery mechanics and fitters	379,211
7241	Electrical mechanics and fitters	649,908
8000	Plant and machine operators and assemblers	276,242
8100	Stationary-plant and related operators	1,728
8110	Mining- and mineral-processing plant operators	21
8111	Mining-plant operators	117
8112	Mineral-ore- and stone-processing-plan operators	9,808
8113	Well drillers and borers and related workers	13,032
8120	Metal-processing-plant operators	478,867
8121	Ore and metal furnace operators	1,816
8122	Metal melters, casters and rolling-mill operators	14,448
8130	Glass, ceramics and related plant operators	3,035
8131	Glass and ceramics kiln and related machine operators	54,347

8152	Chemical-heat-treating-plant operators	1,268
8160	Power-production and related plant operators	2,072
8161	Power-production plant operators	10,974
8162	Steam-engine and boiler operators	61,041
8281	Mechanical-machinery assemblers	34,737
8310	Locomotive-engine drivers and related workers	276,486
8311	Locomotive-engine drivers	67,627
8312	Railway brakers, signallers and shunters	124,194
8340	Ships' deck crews and related workers	89,924
9140	Building caretakers, window and related cleaners	135
9141	Building caretakers	1,462,277
9300	Labourers in mining, construction, manufacturing and transport	42,620
9310	Mining and construction labourers	149,570
9311	Mining and quarrying labourers	2,676
9313	Building construction labourers	688,739
9320	Manufacturing labourers	837,247
9322	Hand packers and other manufacturing labourers	257
9333	Freight handlers	n.r.*

*n.r., not reported (number of observations <5)

Table S7. Incidence rates of asbestosis during follow-up per 100,000 person-years

1977-79	1980-84	1985-89	1990-94	1995-99	2000-04	2005-09	2010-15
2.9	1.6	3.1	7.0	4.0	2.7	2.2	2.0

Supplementary figure S1. Establishment of the study population.

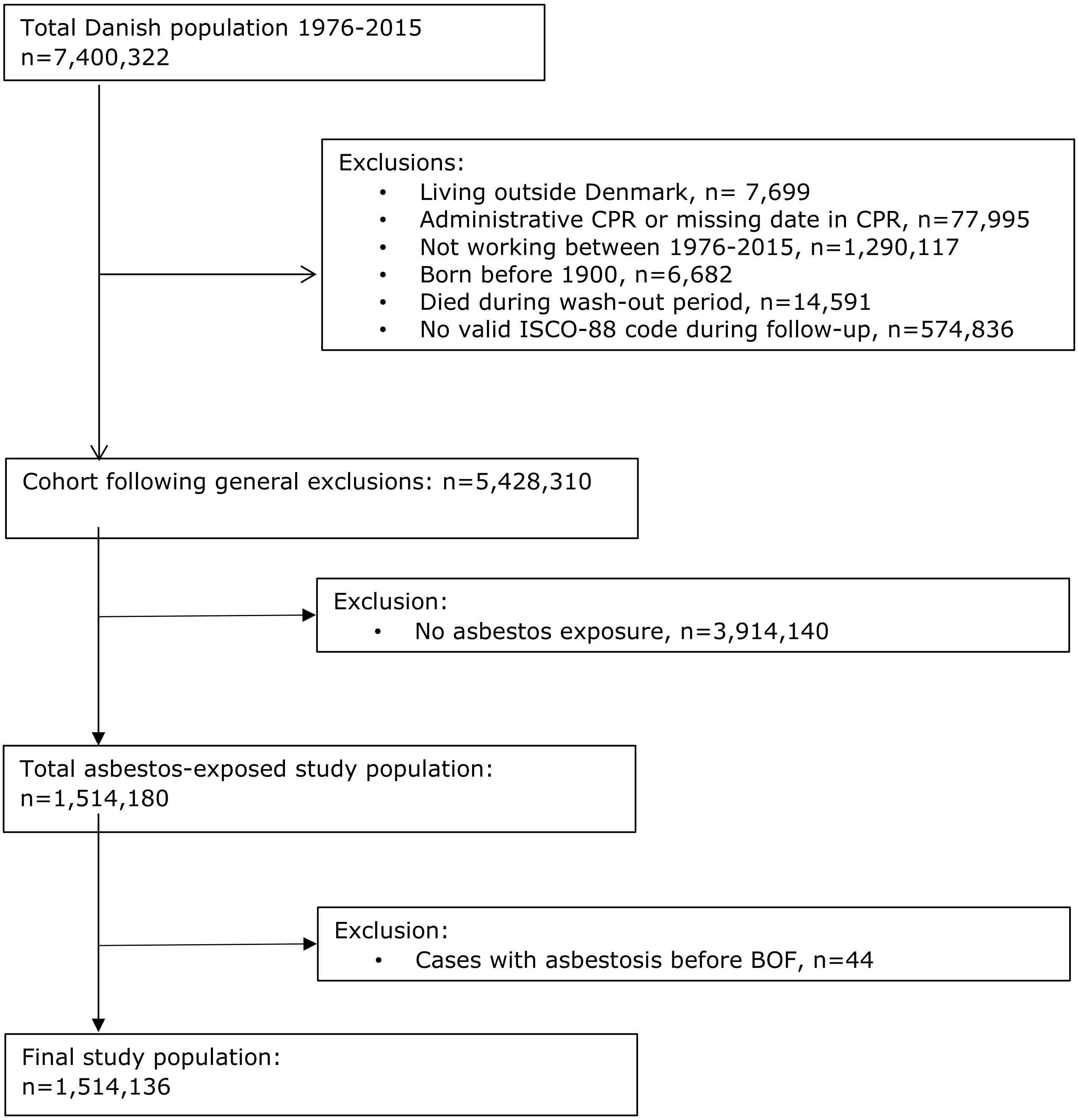

Supplementary references

E1. Lynge E, Sandegaard JL, Rebolj M. The Danish National Patient Register. Scand J Public Health. 2011;39(7 Suppl):30-3.
2. Kildemoes HW, Sørensen HT, Hallas J. The Danish National Prescription Registry. Scand J Public Health. 2011;39(7 Suppl):38-41.
3. Department of Economic and Social Affairs. International Standard Industrial Classification of All Economic Activities (ISIC), Rev. 4. United Nations. 2008.
4. Flachs EM, Bondo Petersen S, Kolstad HA, et al. Cohort Profile: DOC*X: a nationwide Danish occupational cohort with eXposure data - an open research resource. International journal of epidemiology. 2019.
5. Peters S, Vermeulen R, Portengen L, et al. SYN-JEM: A Quantitative Job-Exposure Matrix for Five Lung Carcinogens. The Annals of Occupational Hygiene. 2016;60(7):795-811.
6. Sunyer J, Kogevinas M, Kromhout H, et al. Pulmonary ventilatory defects and occupational exposures in a population-based study in Spain. Spanish Group of the European Community Respiratory Health Survey. American journal of respiratory and critical care medicine. 1998;157(2):512-7.
7. Bondo Petersen S, Flachs EM, Prescott EIB, et al. Job-exposure matrices addressing lifestyle to be applied in register-based occupational health studies. Occupational and environmental medicine. 2018;75(12):890-7.
8. Bonde JPE, Flachs EM, Madsen IE, et al. Acute myocardial infarction in relation to physical activities at work: a nationwide follow-up study based on job-exposure matrices. Scand J Work Environ Health. 2020;46(3):268-77.
9. Lee CT, Feary J, Johannson KA. Environmental and occupational exposures in interstitial lung disease. Curr Opin Pulm Med. 2022;28(5):414-20.
10. Bae W, Lee CH, Lee J, et al. Impact of smoking on the development of idiopathic pulmonary fibrosis: results from a nationwide population-based cohort study. Thorax. 2022;77(5):470-6.
11. Raghu G, Wilson KC, Bargagli E, et al. Diagnosis of hypersensitivity pneumonitis in adults: An official ATS/JRS/ALAT clinical practice guideline. American Journal of Respiratory and Critical Care Medicine. 2020;202(3):E36-E69.
12. Camus P, Fanton A, Bonniaud P, et al. Interstitial lung disease induced by drugs and radiation. Respiration; international review of thoracic diseases. 2004;71(4):301-26.
13. Fischer A, du Bois R. Interstitial lung disease in connective tissue disorders. Lancet (London, England). 2012;380(9842):689-98.
